# Polymerase Chain Reaction (PCR) Profiling of Extensively Drug-Resistant (XDR) Pathogenic Bacteria in Pulmonary Tuberculosis Patients

**DOI:** 10.7759/cureus.61424

**Published:** 2024-05-31

**Authors:** Avantika Ranganathan, Durai Singh Carmelin, Raman Muthusamy

**Affiliations:** 1 Center for Global Health Research, Saveetha Medical College and Hospitals, Saveetha Institute of Medical and Technical Sciences, Saveetha University, Chennai, IND

**Keywords:** pcr (polymerase chain reaction), anti-tb drugs, antibiotic resistance, pulmonary tuberculosis, extensive drug resistance (xdr)

## Abstract

Introduction

Pulmonary tuberculosis (TB) remains a global health concern, exacerbated by the emergence of extensively drug-resistant (XDR) strains of *Mycobacterium tuberculosis*. This study employs advanced molecular techniques, specifically polymerase chain reaction (PCR) profiling, to comprehensively characterize the genetic landscape of XDR pathogenic bacteria in patients diagnosed with pulmonary TB. The objective of the study is to elucidate the genes that are associated with drug resistance in pulmonary TB strains through the application of PCR and analyze specific genetic loci that contribute to the development of resistance against multiple drugs.

Materials and methods

A total of 116 clinical samples suspected of TB were collected from the tertiary healthcare setting of Saveetha Medical College and Hospitals for the identification of MTB, which includes sputum (n = 35), nasal swabs (n = 17), blood (n = 44), and bronchoalveolar lavage (BAL) (n = 20). The collected specimens were processed and subjected to DNA extraction. As per the protocol, reconstitution of the DNA pellet was carried out. The reconstituted DNA was stored at -20 °C for the PCR assay. From the obtained positive sample specimens, XDR pulmonary TB specimens were focused on the targeted genes, specifically the *rpoB* gene for rifampicin resistance, *inhA*, and *katG *gene for thepromoter region for isoniazid resistance.

Results

Out of a total of 116 samples obtained, 53 tested positive for pulmonary TB, indicative of a mycobacterial infection. Among these positive cases, 43 patients underwent treatment at a tertiary healthcare facility. Subsequently, a PCR assay was performed with the extracted DNA for the target genes *rpoB*, *inhA*, and *katG*. Specifically, 22 sputum samples exhibited gene expression for *rpoB*, *inhA*, and *katG*, while nine nasal swabs showed expression of the *rpoB* and *inhA *genes. Additionally, *rpoB *gene expression was detected in seven blood specimens, and both *rpoB* and *inhA *genes were expressed in five BAL samples.

Conclusion

The swift diagnosis and efficient treatment of XDR-TB can be facilitated by employing advanced and rapid molecular tests and oral medication regimens. Utilizing both newly developed and repurposed anti-TB drugs like pretomanid, bedaquiline, linezolid, and ethionamide. Adhering to these current recommendations holds promise for managing XDR-TB effectively. Nevertheless, it is significant to conduct well-designed clinical trials and studies to further evaluate the efficacy of new agents and shorter treatment regimens, thus ensuring continuous improvement in the management of this challenging condition.

## Introduction

Tuberculosis (TB) is an infectious disease that can be contagious. The disease TB is caused by the bacterium* Mycobacterium tuberculosis* (MTB), which is well known as a virulent pathogen.* M. tuberculosis*, which primarily targets the lungs, results in the characteristics and symptoms of pulmonary TB. However, the bacterium has the capability to affect other organs such as lymph nodes, the brain, kidneys, spine, and tissues, expanding the range of potential manifestations beyond the respiratory system, and that leads to the condition called extrapulmonary TB [1]. Despite achieving a microbiologic cure, approximately half of the individuals who survive TB experience some degree of persistent pulmonary TB [2].

In 2022, it was suggested that 410,000 people (95%) are an estimated number of individuals developing multidrug-resistant TB (MDR-TB) or rifampicin-resistant TB (RR-TB). However, approximately 175,650 individuals were found to have been diagnosed and received treatment. This highlights the critical gap in identifying and addressing this drug-resistant form of TB [3]. Furthermore, an additional complex condition was found: isoniazid resistance, a factor contributing to RR-TB [4]. Overall resistance rates for isoniazid were around 16%, which can vary significantly between newly diagnosed and previously treated patients. This condition emphasizes the need for multifaceted strategies to combat the growing threat of drug-resistant TB (DR-TB). The persistence of DR-TB has consistently posed a threat to the effective control of TB. It is crucial to achieve early diagnosis and ensure the timely and comprehensive treatment of DR-TB to prevent further spread [5]. However, the early diagnosis of pulmonary TB infection is critical for controlling TB. One of the main reasons for this factor is said to be the repetition of visits to healthcare-level hospitals and nonspecific antibiotics [6]. Indeed, there is an intensive effort toward the advanced development of innovative medical technologies aimed at preventing, diagnosing, and treating TB. The collective effort is essential in order to enhance our capabilities in addressing the challenges posed by TB effectively [7-10].

Polymerase chain reaction (PCR) has become an increasingly employed technique for the more sensitive and rapid diagnosis of various infectious diseases, including TB. In recent years, substantial progress in PCR technologies has occurred, particularly with the advent of PCR. Multiplex PCR offers several notable advantages, such as reduced turnaround times, automated procedures leading to decreased hands-on time, and a lowered risk of cross-contamination. These advancements contribute to the efficiency and reliability of the diagnostic process for infectious diseases like TB [11,12]. Hence, it is crucial to consider not only the results of laboratory assays but also the clinical aspects when implementing a new methodology, such as the PCR assay. The objective of the study was to determine the prevalence of pulmonary TB and detect the multidrug resistance of specific strains of pulmonary TB infections using a PCR assay, paving the way for subsequent analysis and treatment steps.

## Materials and methods

This study was conducted at Saveetha Medical College and Hospitals, Saveetha Institute of Medical and Technical Sciences, Saveetha University, Chennai, India.

Materials and chemicals

Ziehl-Nielsen stain, blood agar, Löwenstein-Jensen medium, and biochemical reagents were purchased from HiMedia Laboratories Private Limited (Mumbai, India). The strains of extensively drug-resistant (XDR) pulmonary TB were obtained from the Department of Microbiology at Saveetha Medical College and Hospitals Laboratory.

Sample collection

Clinical specimens of sputum, nasal swabs, blood, and bronchoalveolar lavage (BAL) were collected from patients suspected of pulmonary TB following standardized aseptic techniques. Samples were stored at an appropriate temperature and transferred to the laboratory for further analysis. The sample collection mentioned above was approved by the Institutional Review Board Ethics Committee of Saveetha Medical College and Hospitals (IRB No. 112101168) in accordance with relevant regulatory guidelines.

Methodology

PCR Assay of XDR Pulmonary TB DNA

A hospital-based clinical study was carried out on the patients who attended Saveetha Medical College, Chennai, from August 2023 to February 2024. A total of 116 cases were suspected of TB; out of those, 53 individuals were suspected of pulmonary TB. The collected samples include sputum (n = 35), nasal swabs (n = 17), blood (n = 44), and bronchioalveolar fluid (n = 20). All individuals gave their consent to participate in the study. The processing of the sample differed according to the different specimen types. Standard N-acetyl cysteine sodium hydroxide (NaOH) decontamination was performed for non-sterile samples [13,14]. Symptoms like chronic cough, fever, and weight loss for a minimum of two weeks were considered for the evaluation of TB. Based on the clinical symptoms, the patients underwent screening by bacterial culture and imaging examination. Based on the protocols followed, the study employed conventional methods like acid-fast bacilli staining. The extraction of DNA and PCR assays were performed based on Van Der Zanden et al. and Davis et al. studies, respectively, with minor modifications [15-17]. The collected data were analyzed by Microsoft Excel (Microsoft Corporation, Redmond, Washington, United States). The sensitivity and specificity and their respective confidence intervals were calculated [18]. The statistical significance of these findings was compared to the reference standard, and a p-value <0.05 was determined by the chi-square test. The primers for the target genes *rpoB*, *inhA*, and *katG *and their results were interpreted as follows (Table 1).

**Table 1:** Forward and reverse primers for the multiplex PCR amplification F, forward primer; PCR, polymerase chain reaction; R, reverse primer Source: Yang et al. [19], Sinha et al. [20]

Gene	Primer sequence (5′-3′)	PCR product size	Purpose
rpoB	F:GGTCGGCATGTCGCGGATGG R: TGTATGCGACGGGTGCA-CGTC	270 bp	Rifampicin resistance detection
inhA	F: GCGCGGTCAGTTCCACA R: CACCCCGACAACCTATCG	270 bp	Isoniazid resistance detection
katG	F: ATACGACCTCGATGCCGC R:GCAGATGGGGCTGATCTACG		

## Results

**Figure 1:**
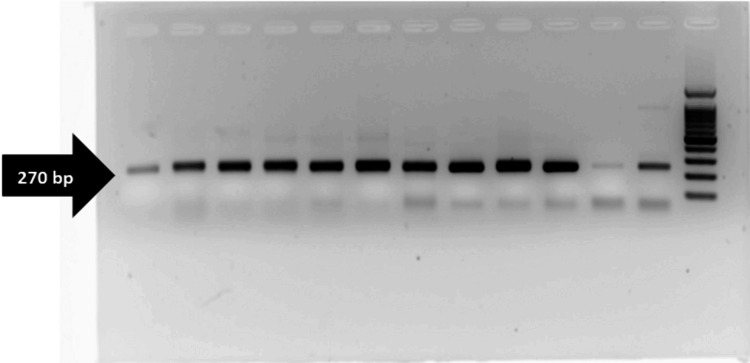
Gel electrophoresis PCR analysis of rpoB, inhA, and katG genes in the 270 bp region Nasal swabs and sputum samples PCR, polymerase chain reaction

**Figure 2:**
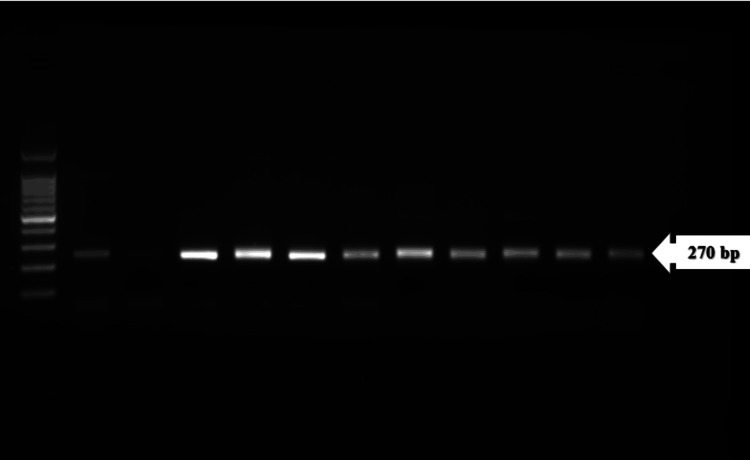
Gel electrophoresis PCR analysis of rpoB, inhA, and katG genes in the 270 bp region Blood and BAL samples PCR, polymerase chain reaction

Out of a total of 116 samples obtained, 63 were diagnosed with non-TB mycobacteria (NTM), while five samples were contaminated and hence excluded from further analysis. Among them, 53 tested positive for smear results indicative of mycobacterial infection. Among these positive cases, 43 patients underwent treatment at tertiary healthcare facilities. Subsequently, a PCR assay for target genes *rpoB*, *inhA*, and *katG *was performed on the extracted DNA from these samples. As a confirmatory method to detect the presence of XDR strains *rpoB*, *inhA*, and *katG*, a gene indicating resistance to rifampicin and isoniazid antibiotics, respectively, was identified in the 270 bp region (Figures 1, 2). Specifically, 22 sputum samples were detected with the target genes *rpoB*, *inhA*, and *katG*, while nine nasal swabs were identified with *rpoB *and *inhA *genes. Additionally, the *rpoB *gene was detected in seven blood samples, and both *rpoB *and *inhA *genes were found in five BAL samples (Table 2).

**Table 2:** Types and the total number of samples collected and their resistant pattern BAL, bronchoalveolar lavage; R%, resistance in percent; TB, tuberculosis

Types of samples collected	Total number of samples collected (n)	Number of non-TB samples	Number of samples positive for pulmonary TB (n)	Number of samples not detected for resistant *rpoB*, *inhA*, and *katG *genes	Positive for the rifampicin-resistant gene *rpoB* (n) (R%) (n = 43) R = 81%	Positive for isoniazid resistant gene *inhA* (n) (R%) (n = 43) R = 81%	Positive for isoniazid resistant gene *katG* (n) (R%) (n = 43) R = 81%
Sputum	35	18	23	1	16 (53.3%)	4 (50%)	2 (40%)
Nasal swabs	17	16	12	3	7 (23.3%)	2 (25%)	-
Blood	44	14	10	3	4 (13.3%)	-	3 (60%)
BAL	20	15	8	3	3 (10%)	2 (25%)	-
Total	116	63	53	10	30 (56.6%)	8 (15%)	5 (9.4%)

The PCR amplification targeting genes *rpoB*, *inhA*, and *katG *was conducted on samples obtained from patients diagnosed with NTM and exhibiting smear results indicative of mycobacterial infection. A total of 43 patients who tested positive for smear results underwent treatment in tertiary health care facilities. Among the sputum samples (n = 22), *rpoB*, *inhA*, and *katG* genes were detected in all samples. This suggests a high prevalence of mycobacterial infection in the buccal cavity of the patients included in the study. For nasal swabs (n = 9), *rpoB*, *inhA*, and *katG *genes were observed in all samples tested, indicating the presence of mycobacterial DNA in the respiratory tract. However, the *katG *gene was not detected in any of the nasal swab samples. In blood samples (n = 7), the *rpoB* gene was detected in all samples, while the *inhA* gene was absent (Table 3). This suggests a potential dissemination of mycobacterial infection is present in the lower respiratory tract of these patients, with a high likelihood of active infection. Overall, the PCR assay results highlighted the diverse distribution of mycobacterial DNA across different sample types, emphasizing the importance of comprehensive diagnostic approaches in the management of NTM infections.

**Table 3:** Results of PCR amplification targeting genes rpoB, inhA, and katG in different sample types BAL, bronchoalveolar lavage; PCR, polymerase chain reaction

Sample type	*rpoB *gene	*inhA* gene	*katG *gene
Sputum (n = 22)	22	22	22
Nasal swabs (n = 9)	9	9	0
Blood specimens (n = 7)	7	0	0
BAL (n = 5)	5	5	0

## Discussion

The combat against TB has become complex due to the increasing MDR or XDR strains of MTB. This development is due to the mutations caused by the specific genes present in the bacterial chromosome. These mutations can occur in genes like *rpoB*, *katG, inhA*, *pncA*, *embB*, *gyrA*, and *gyrB *[21]. Each of the genes has a vital role in the susceptibility of the bacteria to various anti-TB medications. These mutations essentially render the drugs ineffective by complicating treatment strategies and global control efforts. Hence, early and accurate diagnosis is crucial for effectively managing patients with DR-TB to prevent the further spread of these strains, which involves rapid identification of specific chromosomal gene mutations within MTB that are known to cause resistance to different TB medications. Regular monitoring of the prevalence of these mutations in the areas with high TB impacts. Therefore, there might be proactive identification and addressing of the challenges of DR-TB transmission [22]. Implementation of new medications is an essential step among the various therapeutic strategies. Based on the data from the NIX-TB trial conducted in South Africa, the Food and Drug Administration in August 2019 approved the recommendation of incorporating pretomanid alongside bedaquiline and linezolid, with the option to include moxifloxacin. The addition of pretomanid simplifies the treatment process [23,24]. Additionally, ethionamide has been recognized for its effectiveness in treating drug-susceptible TB, making it a valuable option for the recently introduced six-month regimen for this form of TB as well [25].

Molecular diagnostic techniques play an indispensable and indisputable role in the diagnosis of TB due to their precise nature [26]. In resource-limited settings, the development and utilization of molecular assays offer a cost-effective and pragmatic alternative for TB diagnosis. Molecular assays such as PCR target specific sequences of the TB bacteria, allowing for sensitive and rapid detection of the pathogen. By customizing these assays to the local conditions, including equipment availability, technical expertise, and infrastructure constraints, healthcare providers can optimize their utility in resource-limited settings [27].

TB prevalence differs significantly between urban and rural areas, as urban areas show lowered TB rates with a moderate yearly increase, while rural areas have higher TB rates with a slower annual rise [28]. This study was initiated in 1968 as a vaccine trial by ICMR-NIRT in Thiruvallur, Chennai, India [29]. In accordance with the 2021 survey findings, the incidence of pulmonary TB, with a specific focus on the Thiruvallur district, reveals notable prevalence rates. Within the population of 100,000 individuals, the survey identified 307 cases demonstrating bacteriological positivity [28]. This data underscores the significant burden of XDR pulmonary TB within the specified region, highlighting the urgency for targeted interventions and public health strategies to reduce the impact and prevalence. In our study, we collected a diverse array of samples, including sputum, blood, BAL, and acetic fluid, despite encountering certain logistical constraints in the tertiary healthcare setting of Thiruvallur district. These samples were then subjected to multiplex PCR amplification aimed at detecting specific genes associated with resistance to first-line antibiotic drugs. This approach allowed us to comprehensively assess antibiotic resistance patterns across different types of specimens, providing valuable insights into the molecular epidemiology of DR-TB.

The rise of multidrug resistance or extensively drug resistance isolates in specific geographic regions highlights the need for further research to focus on the specific gene mutations driving this epidemic. Furthermore, the growing trend of resistance to rifampicin, isoniazid, and the emergence of MDR-TB in both new and previously treated patients underscores the importance of improved patient management strategies. By focusing on better management, we can potentially prevent the further evolution of DR-TB strains.

Limitations

Tracking the frequency of the most common target genes, such as *rpoB*, *inhA*, and *katG*, among rifampicin- and isoniazid-resistant isolates would be a valuable indicator for the development of TB diagnostics and treatment regimens. This would also prevent further development and reduce the risk of MDR/XDR-TB drug resistance patterns in patients receiving the various treatment strategies. In the above scenario, the present study findings, although small (n = 116), would add value in developing appropriate diagnostics and stewardship programs that guide clinicians at the appropriate time.

## Conclusions

DR-TB is considered a major public health concern worldwide, with mutations in MTB leading to phenotypic resistance to antimicrobials currently available and used. The increasing frequency of the occurrence of XDR-TB in clinical samples led to a retardation in the progress of various combinations of therapeutic strategies to control the MTB. Hence, it is critical to detect the various drug-resistant genes in the clinical sample using a PCR-based assay to formulate and effectively use the currently available anti-TB drugs, either singly or in combinations, to achieve the elimination of TB. The present study findings revealed a significant presence of resistant genes such as *rpoB *(56.6%), *inhA *(15%), and *katG* (9.4%), indicating extensively resistant strains to rifampicin and isoniazid prevailing in the cases in tertiary health care hospitals. Based on the above findings, necessary modifications in the therapeutic regimen of antibiotics may be advised, leading to the development of a stewardship program.
